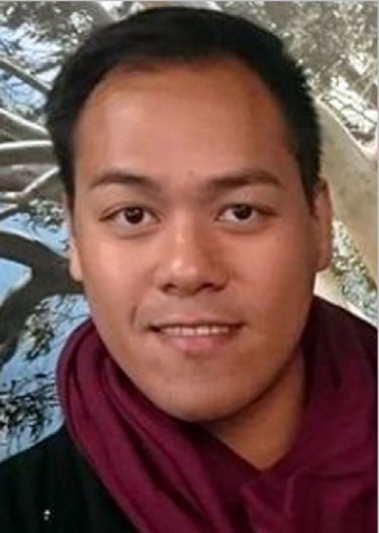# Six Laws of Open Source Drug Discovery

**DOI:** 10.1002/cmdc.201900565

**Published:** 2019-10-15

**Authors:** Matthew H. Todd

**Affiliations:** ^1^ School of Pharmacy University College London 29–39 Brunswick Square WC1N 1AX London UK

## Abstract

**Six to swear by**! Society needs effective and affordable medicines. We currently have at our disposal essentially one system to discover and develop drugs, and there are many areas where this system struggles to deliver, for example to combat antimicrobial resistance, or tropical diseases, or dementia. It is sensible to cultivate alternative, competing approaches to drug discovery and development. A genuinely new alternative is to open up the entire research cycle, abandoning secrecy altogether. This “open source” approach has now been trialed and the lessons learned distilled to six laws of operation that help to clarify working practices. This article examines and explains those laws, which can be adopted by anyone wishing to create medicines using an inclusive, public process.

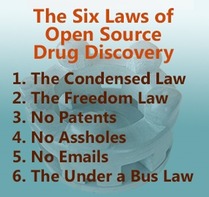

## 1 Open Source Applied to Drug Discovery

Creating and developing new medicines requires a stream of innovations. Many of these are in the technical side of our discipline: methods in organic synthesis, assay technologies, machine learning, or approaches based in fundamental biology. Some innovations arise in allied disciplines of economics, or law. But in parallel we must try to notice when our work patterns are constrained by social structures that may limit our abilities to function most effectively. Are we going about things in the right way? Are we innovating *in the way that we work*?

There are large‐scale initiatives in pharma trying to address this under the banner of “open innovation”.^1^ The term is nebulous, but there is typically some re‐orientation of a company to be more outward‐facing—to place problems in the public domain and attempt to broaden the net of expertise in order to solve those problems faster. The broad range of such initiatives means that while some are highly valuable, others are branding exercises. Either way they will in general not change the way we work: just as for secretive, inward‐facing endeavours, participants in open innovation projects typically work in closed groups, revealing only that which is felt worth sharing. The open part of open innovation is the shared problem, but not necessarily the proposed, and attempted, solutions.

Examples of an alternative way of working are all around us, but largely viewed as insufficiently serious for science. *Wikipedia* has transformed the way knowledge is curated, aggregated, and shared, yet is probably the tool that scientists deny using most frequently. The software that underpins the internet was built using a method similar to that behind *Wikipedia*, specifically that the details can be seen by anyone and people can work together openly to improve the content or take it in a new direction. There is no appetite for secrecy or silos because those stifle our ability to access the best ideas, and why would you want to do that? This method is ***open source***, distinct from open innovation and to be distinguished from open access, which term refers to our ability to read papers—valuable, but not the transformative feature of initiatives like *Wikipedia*. In open *source* the community participates in peer‐reviewed public work. Depending on the exact licence of the project, one will have high levels of freedom to act. Crucially, one may act while the work is happening, rather than merely after it is complete. You are a player, not an observer. There is cooperation towards goals that operates alongside an open competition in how work is done, i.e., collaboration within a rather brutal, open arena of expertise.

Over the last 15 years, I have worked with large numbers of people who are interested in open source in drug discovery because the approach promises to be able to solve problems in exactly the way that the pharma industry, if acting alone, cannot. If one values competition, as I suspect all scientists do, then a competition of approaches must be seen as healthy. If open source can compete with proprietary methods in a commercial space as valuable as telecommunications (i.e., the projects leading to Android *vs*. iOS) can the same competition of ideas benefit another commercially‐important area: drug discovery?

I strongly suspect it can. The answer depends on the disease. But I am running this experiment with others because the answer is not yet clear.

This article is intended to clarify key terms. One cannot set off for the moon without some basic principles of operation, and vagueness can be worse than nothing. We ought to be clear what “open source drug discovery” means. When I started in this endeavour the term was aspirational, but we have now run several successful projects with a clear and unified set of principles, and I would like to highlight these because they are useful. Operations have been distilled to six laws that have held strong in the real world and which are the subject of this article.

## 2 How the Laws Came About

As with any endeavour designed to elicit maximum inclusion and productivity, there is a fine line to navigate between freedom to operate on the one hand and a sense of order and unified purpose on the other. Yuval Noah Harari makes a nice point in his book *Sapiens* that corporations exist beyond the physical – that they may possess a lasting identity without any permanent assets.^2^ It is the same with open source initiatives. The lack of a tangible “thing” or “building” remains one of the most challenging ideas for those coming to an open source project for the first time, yet the fluidity of its structure and constitution provides the kind of resilience you observe when trying to spear a shoal of fish. “What is an open source project?” is a complicated question, but a short form answer might be “people, and their enthusiasm for a shared public mission”.

The Six Laws attempt to capture some of this structure, but were not set up *a priori*. They arose from the first project I ran in an open way: the search for a robust route to an enantiopure version of the world′s most widely used anthelmintic, praziquantel.^3^ The need for such an improvement had been explicitly mentioned as a research priority by the tropical disease research division of the World Health Organisation. To solve it, I proposed some chemically elaborate approaches and placed them online in an attempt to kick‐start an open approach to solving this chiral switch. My intention was to mimic the hive mind that was, in late 2004, having a major impact on the construction of new software and *Wikipedia*. The project became busy once we were modestly funded. At this point reality collided with the grandeur of grant proposals and the scientific approach we were taking changed in response to precisely the expert collective intelligence I had hoped for, freely given and mostly from the private sector. The solution emerged swiftly and was solid. The science was unquestionably accelerated through the inputs of well‐qualified strangers.

Over a dinner in Cape Town hosted by Kelly Chibale I got talking with Tim Wells, the CSO of the Medicines for Malaria Venture (MMV), who asked the obvious question: could we use the same approach to the discovery not of better chemical routes, but of new chemical entities? The conversation went back and forth until we decided that conversation can only take people so far, and it is sometimes better to learn by doing. Open Source Malaria (OSM, initially called Open Source Drug Discovery for Malaria) was born from that conversation and the invaluable wisdom of the MMV team of Jeremy Burrows and Paul Willis. I realized that this might turn into a larger endeavour: there were no such initiatives anywhere else. Genomics collaborations, such as the Human Genome Project, had pioneered the sharing of data sets with clear commercial potential, downstream. There continues to be a great deal of impactful research into open source tools in cheminformatics.^4^ The Open Source Drug Discovery project in India was, despite its name, operating a crowdsourcing initiative as opposed to something that was open source.^5^ The Structural Genomics Consortium were pioneering the bold sharing of chemical probes (not drugs or their analogs) and were not proposing the sharing of the full research cycle that led to them.^6^ We were proposing something different—secrecy‐free creation of new medicines.

To minimize confusion, it was important to capture the core principles of OSM in a way that would be simple to understand for potential incomers and to ensure those joining in knew the level of mischief they would be getting into. So on July 25^th^ 2011, led by what I felt to be the most important lessons learned from the WHO project, I wrote down the ‘Six Laws’ of OSM.^7^ The list always brings out the most phone cameras when I speak about open source drug discovery, an observation that has led to this article. What I find remarkable is how well these Laws have stood the test of time, holding essentially unaltered for 8 years and guiding the involvement of, to date, over 300 people on the four OSM campaigns. The Laws can apply equally well to the next campaigns that I, or others, create under OSM′s banner or to similar projects in other areas of drug discovery that people might want to run.^8^ The Laws are intended to guide behaviour, to free people to act to the best of their ability within a framework that promotes a distinctive discovery process. They do not hasp and hoop the contributors, and can be changed in the future if they are found to be faulty.

The first three Laws clarify day‐to‐day operations. The others are more subtle, big‐picture concepts.

## 3 The Laws

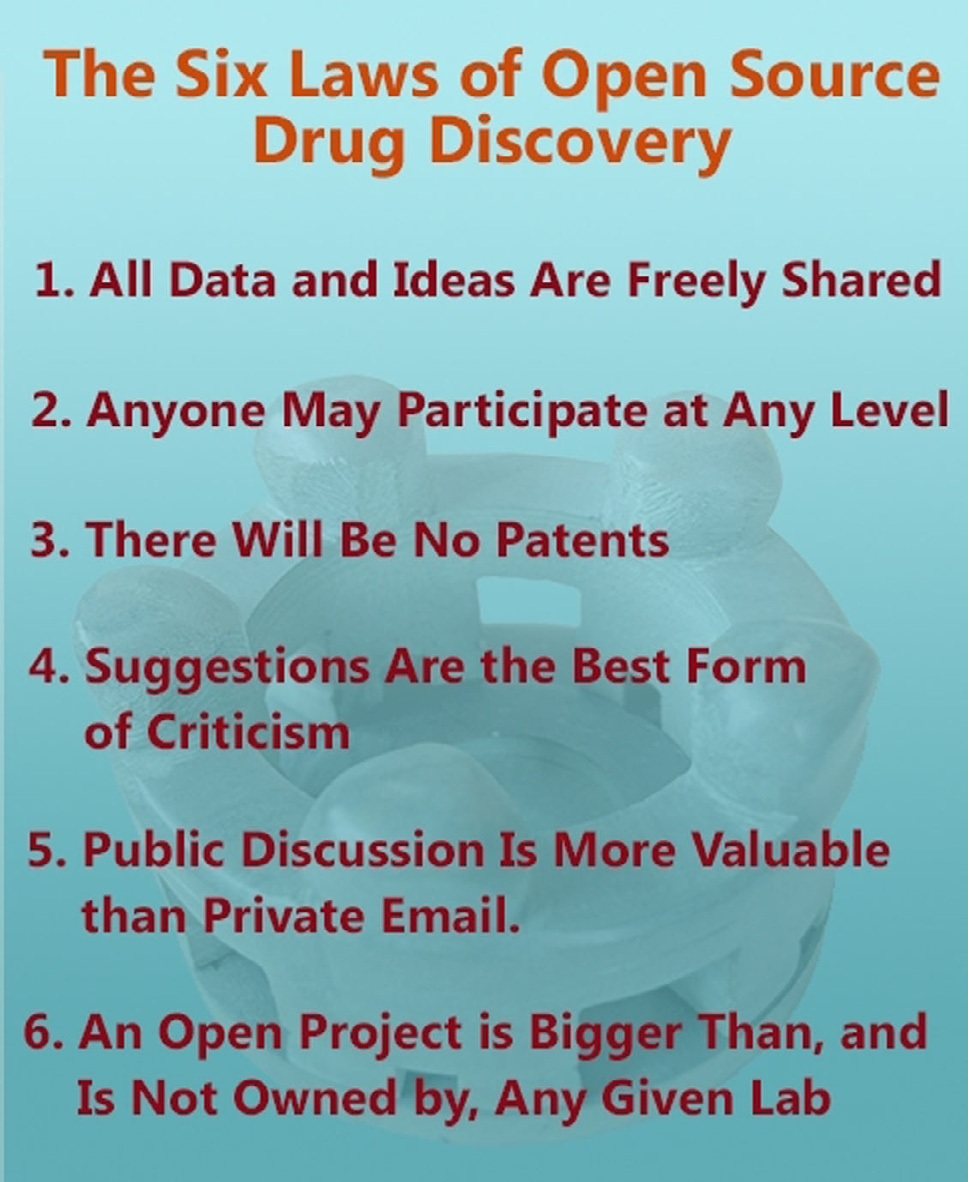

## 1) All Data and Ideas Are Freely Shared.

This is a deceptively short Law that we ought to unpick if we are not to miss some essential features. We might call it “The Condensed” law. When experiments are performed, those need to be recorded in a laboratory notebook (obviously), and that notebook (wherever it is) needs to be available to read in its entirety. This means it needs to be online and, to be of any practical use, not behind a password wall. It is remarkable how many academic scientists are still using paper lab books that reside on desks, as if in homage to Leonardo da Vinci. We have not the space to cover the subject of notebooks here (typically “Electronic Laboratory Notebooks” or ELNs),^9^ but given peoples′ propensity to have opinions, we cannot proscribe a particular ELN (which would be a distraction) and should instead focus on the core FAIR principles of findability, accessibility, interoperability, and reproducibility.^10^ It is important that there is an electronic record and that we can find it. An open source ELN would be desirable. Some exist, but they are not perfect, or well‐supported.^11^ It is not as though a commercial ELN is a derogation of the first law, if that solution allows all the data to be seen, exported and re‐used (so, open file formats are good). We should just find a solution that works for the scientist or the team and ensure the contents are openly available. In OSM there are many examples that aim for this;^12^ some entries may fail to live up to all the formal requirements, and we can only try.

The lab notebook needs to contain all the project data. That means the TLCs, the NMR data (i.e., the file, not just a PDF), everything. You may say “but who will ever read such a thing?” to which the answer is “who ever reads a lab notebook cover to cover?” There must be a primary repository of information from which everything else comes. Without full disclosure we break an important line of trust between participants.

The word “ideas” is included in the Law since it is important not only what has been done, but what is going to be done. Naturally, ideas may be better placed in a location more suited to focused discussion of objectives than an ELN. For some time we struggled with this, and have found an efficient solution on a widely‐used software development platform called GitHub, which has an “issue tracker” function (essentially a place to discuss, and resolve, smaller problems).^13^ Ideas can be mooted, discussed, opened and closed, assigned to individuals for action, and incorporated into other discussions. Conclusions and decisions can be folded into a summary wiki describing where the project is up to. Thus forward planning is essential, but needs functionality not present in ELNs.

The words “freely shared” are included because people should not feel inhibited in sharing everything they can. But there is the other, more substantial, meaning of “free” which brings up the subject of licences (the word “free” has a particularly complicated nuance in software).^14^ When things are shared, what are the terms? There are many licences for open source software projects, none of which quite work for us because drug discovery involves tangible objects, ideas, data and assay platforms. To allow for an easily‐understandable structure in OSM we adopted the Creative Commons CC‐BY licence (used by *Wikipedia*) meaning you can use anything you want in the project, including for the purposes of making money, provided you cite the project.^15^ This works as an interim, until we come up with something more robust. Note that there is no “viral” aspect to the licence: if you use something in OSM, you need not share your work under the same terms. While this would be fundamentally desirable, and is clearly implied by participation in the first place, obligations of this sort might prevent people getting involved for no other reason than people are cautious about constraining their future. So, nothing viral.

The First Law establishes the day‐to‐day way of working. Conduct experiments, keep a comprehensive lab notebook that people can see, and which contains all your data, share it with a clear (e.g., CC‐BY‐style) licence, and be sure to use your conclusions to share your ideas of what should be done next.

## 2) Anyone May Participate at Any Level.

Science is a team sport. There is no point in doing it unless you want to work with others to get things done more quickly, and there is no point in working open source unless you want to work with intelligent strangers. If you want to learn how to play with Lego, or you want to build something big with it, tip the box on the floor and make room for others. If you are going to define at the outset who can participate, then you are looking for a traditional collaboration. If you do not know who might be the best person to work with, go open source. So: allow anyone to participate, and allow them to do anything they want. This second Law, then, is the combined “Freedom and Low‐hierarchy” law.

The words “any level” remind us that people might want to do many different things, from commenting on data to designing a synthesis to actually carrying out an experiment through to starting a whole set of experiments themselves. All of these things have happened in OSM. Senior pharma professionals have run experiments. Cohorts of school children have innovated. It is almost always the case that people have contacted me or others first to ask, in essence, “are you sure, I mean I can just go ahead and do that?” to which the answer is always “yes.” But the words “any level” also refer to the freedom to work across institutional boundaries, or to act in a way that subverts how one may be seen by others. Undergraduates can (and have) debated points of substance with senior professors without rank becoming an issue. Unlike much of the execrable online conversation we may see in our daily lives, the interactions online in a focused science project are refreshingly productive.

The second law means that we are intentionally setting up an arena of ideas and expertise, and entry is unrestricted. The emphasis on inclusiveness is a useful reminder that open source drug discovery is not “anti‐pharma” and indeed has thrived on the expert contributions of many talented scientists from the private sector.

## 3) There Will Be No Patents.

People with experience of the patent system, upon reading the no‐patent Law, will remind me that a patent is an intentionally open statement of achievement, designed to be provided in sufficient detail that someone else may replicate the work. This public statement is made in return for the protections a patent affords the inventor to nurture the commercial development of the invention. Permit me to put a temporary pin in the observation that the system sits on a firm bedrock of protections given to inventors by the state.

People with experience of the patent system are also aware that in many cases a patent provides not a clear declaration of achievement but rather broad legal claims that border on scientific obfuscation. Patents are frustrating to read and reading a new one is the triumph of hope over experience.

The 3^rd^ Law is intended partly for clarity—that patents are out. The law exists not because of the faults with patents themselves. It exists because if one wishes to gain a patent, one cannot have described one′s work and achievements in the public domain. The requirement to conceal is incompatible with open source.

The required secrecy poisons the effectiveness of the research that is upstream, devoiding it of the efficiencies that one might gain through openness. So one cannot adopt the workaround (suggested by many interested in the aim of affordable medicines) of patenting and then licensing out the results. This is a perfectly reasonable thing to do, but it is not compatible with an unrestricted open source R&D community.

This 3^rd^ Law takes us towards a bigger picture view of matters since it begs the question: if you don′t have patents, how are you going to take a drug through to market? Perhaps in some cases this is a moot point, if for example there is not going to be a realistic market or there is a structural problem with obtaining a financial return (as currently plagues the field of drugs to counter antimicrobial resistance). The answer to the question obviously depends on the nature of the relevant disease, but the question is a major one since an effective answer to it would subvert the status quo of essentially all the drug development work currently going on around the world. The answer is complicated and being discussed in many places, with many possible solutions.^16^ Space here prevents us from reviewing them all, but I would like to highlight just one that is of topical interest.

Some time ago I was speculating on the core challenge of commercial development of open work—that one could not “protect” an idea that was already in the public domain.^17^ I was struck by how appropriate it would be that one should be able to demonstrate that an invention works in the field and then be rewarded somehow, after the fact. Particularly if the invention were developed openly, allowing others to benefit from the details of the research along the way. Clearly for such a thing to work economically (i.e., to recoup expenses or reimburse any investors) the inventor would need a form of temporary protection that triggers when a certain point is reached. It turns out I have family history associated with this idea arising from an invention during the industrial revolution, as I have described elsewhere,^18^ and there are examples of related ideas,^19^ but to translate all this into drug discovery terms one would need, not a patent, but a different temporary exclusivity granted by the state that allows some level of cost recovery (I am retrieving my earlier pin at this point). This might sound outlandish to those focused too much on patents, but it should not because it exists already, in the form of various flavours of regulatory data exclusivity available to drug inventors and intended to protect them in just this way. The idea has been mooted as compatible with open source approaches^20^ and has now been excitingly instantiated in a real company using this idea in its operations, M4K Pharma.^21^ Open research leading to an engaged community leveraging guarantees arising from existing regulatory arrangements as the business model. This is an exciting idea that we can try out in the coming years, and one that anyone should welcome into the chocolate box of competing ideas that we surely need if we are serious about trying genuinely new things.

## 4) Suggestions Are the Best Form of Criticism

This is the “no asshole” rule that simply reminds us all to be constructive when being critical. Open source requires a potentially confronting playing field of competence in which it is perfectly possible for an undergraduate to correct a seasoned academic in a permanent public record. This structure needs to be embraced, but the publicly‐viewable nature of the record of work is one of the concerns I hear from senior academic colleagues: “What if we make a mistake?” There are two parts to such an objection:

a) Data could be wrong, and people may waste their time. As an objection to openness, this is mostly a phantom worry. Data can always be wrong, and people can always be led down unproductive paths. We are all aware of clear, recent examples that make us worried about reproducibility in the academic literature.^22^ An advantage of an open lab notebook is that the level of uncertainty can be laid out clearly (if, for example, a fashionable positive result has been obtained against a background of nine unfashionable negatives), and data can surfaced with a label saying “PRELIMINARY DATA (DRAGONS)” which is not a level of disclosure one frequently sees in high impact factor journals.

b) Data could be wrong, and we may look foolish. This is possible, but I suspect this, too, is a phantom. If doing science teaches us anything it is to be humble before the experiment. As humans we try to see truth by looking at the error‐prone flickering images on the wall of the cave. It is natural to make mistakes, and good scientists forgive the mistakes of others. Yet at the same time, the need to work openly and in real time requires the best of us—the best‐kept record, the most careful conclusions, the best‐prepared arguments, much as we vacuum and tidy before people come round for dinner. To do this “live”—to manage complexity, interconnected ideas, and hypotheses “live and online”—is something that is thrilling to do and, I hope, for the public to watch. It captures just the sorts of real ups and downs of science that we see in the best science dramas and does not sanitize the details in the way that must happen for much of the science communication we might witness on the TV, for example. We learn quickly from our own mistakes and those of others, and it is a shame if pride inhibits this.

Error is inevitable. We need to encourage the brightest among us. These twin facts necessitate our being able to criticize others in public. But one never knows what one′s fellow scientists are dealing with in their offline lives. Law 4 says “Be kind, always”.

## 5) Public Discussion Is More Valuable than Private Email.

A simple “no email” rule. Email is bilateral, or multilateral, but not usually public. If we want to ensure there are no “insiders” we cannot use it.

This is again a law that people can find difficult at first. Frequently, initial contributors will email in ideas or data, and there is a cost of translating these into the public domain, with permission and maybe redaction. As soon as people are comfortable with writing publicly, that cost vanishes. In fact, the GitHub issue tracker (mentioned above) has a nice feature in that it is compatible with email (with an associated but acceptable risk that people may write a private note that appears on the public site). But we do not yet have a perfect solution to light online conversations. Some GitHub threads run to 100 comments or more and become a little cumbersome if nobody digests them into a smaller number of active tasks or forks them to fresh conversations, but these problems beset email chains too. People are reticent to write trivially small notes in public, but there are platforms that might suit (such as Slack and Reddit), and the reticence may anyway be a generational issue. But ensuring key conversations are kept relevant and not swamped by contributions can only really be solved by active research coordination, and it is better for such coordination to be in public (rather than email) so that we ensure everyone is up to speed.

## 6) An Open Project Is Bigger Than, and Is Not Owned by, Any Given Lab.

The “Under a Bus” Law. If a project leader ceases to be the leader (encounter with a bus, or, worse, disinterest), it must be possible to continue the research. This law is therefore a quality‐control reminder of the need for Law 1—that all data and ideas need to have been shared for a seamless continuance to be possible. A project′s leader is leader by virtue of their behaviour, not by virtue of a name tag (I am reminded of the tragicomic award of the Sherriff badge to Billy Curtis’ character Mordecai in the Clint Eastwood movie *High Plains Drifter*). Leadership in open source projects (the role of the “benign dictator”) is a fascinating subject in its own right, but the Law here acts to remind us that the project and its outcomes are king. In the first iteration of this Law there were the additional words “The Aim is to Find a Drug for Malaria as Quickly as Possible,” to remind us that all these laws and considerations were there in order to help us leverage the power of a new way of working *in order to develop a medicine to help people enjoy their lives*. If I decide that a certain target or series of molecules is no good then I need to leave open the option that someone else disagrees and needs to be able to build on what I have done, unfettered by my biases and my hoarding of any data.

This Law is also making a subtle point about branding: that if one wishes people to get together to work on something voluntarily then one needs to minimize ownership. It is not about the person, but the project. *Wikipedia* would unquestionably have been less successful if it had been called “Jimmy Wales′ Encyclopaedia”. The irony of my publishing this paper is not unappreciated, but is countered by Law 2, that anyone can take part at any level.

The Law is also about immortality. Ask a coder how to make their code immortal. Back it up to 100 private servers? Place it, etched, in a tomb? No, the answer often given is to open it up. If you need to stop, make sure that what you have done need never be done again, and that it is clear how someone else can build on it later. To achieve this requires digital infrastructure with a commitment to a level of permanence that we are increasingly coming to rely upon (e.g. Pubchem, arXiv). This makes one think of all the drug discovery campaigns that have been pursued in academia and industry that have either not been published at all, or published incompletely, or stopped for strategic rather than scientific reasons and then not shared. It is unsettling to consider all the work we might inadvertently repeat. To the public it ought to be a scandalous waste of resources. This is not to criticize the scientists who stopped projects on solid grounds, but merely to rail against the assumption that nobody else knows better. There are a lot of flags planted in sand that serve only to mark unpublished data where there should instead be trees of data awaiting gardeners.

If the laws resonate with you, then jump in.

Matthew H. Todd

Chair of Drug Discovery

University College London

## Biographical Information

**Mat Todd** was born in Manchester, England. He obtained his PhD in organic chemistry from Cambridge University in 1999, was a Wellcome Trust postdoc at The University of California, Berkeley, a college fellow back at Cambridge University, a lecturer at Queen Mary, University of London, and between 2005 and 2018 was at the School of Chemistry, The University of Sydney. He is now Chair of Drug Discovery at University College London. His research interests include medicinal chemistry, synthetic methodology, and catalysis. He has a significant interest in open science, and how it may be used to accelerate research towards new medicines. He founded and currently leads several open source drug discovery consortia including Open Source Malaria (OSM) which in 2012 won one of three Wellcome Trust/Google/PLoS Accelerating Science Awards. His lab motto is “To make the right molecule in the right place at the right time”, and his students are trying to work out what this means.

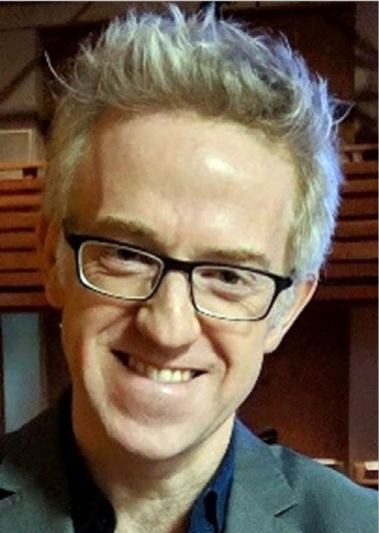

## Biographical Information

**A note from the Editor** Having been involved mostly with biology‐heavy projects when I was an active researcher, I only truly learned about Mat′s passion for open source drug discovery when I joined Wiley and *ChemistryOpen* in 2015, as Mat served (and continues to serve) as an Editorial Board Member of that sister journal. Surprisingly, it was only at last year′s EFMC‐ISMC meeting in Ljubljana, when I first met him in person and heard his excellent talk on his open source projects. That was also the first time I heard of his “Six Laws”, despite these existing for seven years, as he shares in his Guest Editorial. I knew right then that this was an interesting and important message to communicate in print. I am thankful for Mat′s willingness to finally write down his thoughts in this truly insightful article. I am personally a strong believer in the the open movement. I get asked often if open science and open access are the the future of publishing and research, and I say NO. These aren′t the future, because they are the **NOW** of research, and anyone who ignores the significance and speed of the open movement is bound to stagnate and be left in the dust. That said, there is still so much more to be done, and there′s no better time to dive in than now. At the recent European Research and Innovation Days, Prof. Karl Luyben (European Open Science Cloud Chair) mentioned, “If Open Science is the new normal, it would just be called Science.” This is why *ChemMedChem* and Wiley‐VCH are committed to open research and open access more than ever. This is reflected in our support and publication of works from various open consortia, discussions and essays on open science such as this, the development of open and collaborative tools/hubs like https://www.authorea.com/, and the signing of impactful, transformative deals, like the https://www.projekt-deal.de/faq-wiley-contract/ partnership, that don′t just push for OA, but open science in general. As Mat puts it in his essay, Open Science and its Laws are here—**jump in**.

## Biographical Information

**–David Peralta, Editor‐in‐Chief**